# Exfoliation and Noncovalent Functionalization of Graphene Surface with Poly-*N*-Vinyl-2-Pyrrolidone by In Situ Polymerization

**DOI:** 10.3390/molecules26061534

**Published:** 2021-03-11

**Authors:** Suguna Perumal, Raji Atchudan, Thomas Nesakumar Jebakumar Immanuel Edison, Jae-Jin Shim, Yong Rok Lee

**Affiliations:** School of Chemical Engineering, Yeungnam University, Gyeongsan 38541, Korea; suguna.perumal@gmail.com (S.P.); jebakumar84@yu.ac.kr (T.N.J.I.E.)

**Keywords:** exfoliation of graphite, graphene, noncovalent functionalization, in situ polymerization, cyclic voltammetry

## Abstract

Heteroatom functionalization on a graphene surface can endow the physical and structural properties of graphene. Here, a one-step in situ polymerization method was used for the noncovalent functionalization of a graphene surface with poly-*N*-vinyl-2-pyrrolidone (PNVP) and the exfoliation of graphite into graphene sheets. The obtained graphene/poly-*N*-vinyl pyrrolidone (GPNVP) composite was thoroughly characterized. The surface morphology of GPNVP was observed using field emission scanning electron microscopy and high-resolution transmission electron microscopy. Raman spectroscopy and X-ray diffraction studies were carried out to check for the exfoliation of graphite into graphene sheets. Thermogravimetric analysis was performed to calculate the amount of PNVP on the graphene surface in the GPNVP composite. The successful formation of the GPNVP composite and functionalization of the graphene surface was confirmed by various studies. The cyclic voltammetry measurement at different scan rates (5–500 mV/s) and electrochemical impedance spectroscopy study of the GPNVP composite were performed in the typical three-electrode system. The GPNVP composite has excellent rate capability with the capacitive property. This study demonstrates the one-pot preparation of exfoliation and functionalization of a graphene surface with the heterocyclic polymer PNVP; the resulting GPNVP composite will be an ideal candidate for various electrochemical applications.

## 1. Introduction

One of the most studied materials is graphene, where carbon atoms are arranged in a honeycomb lattice structure [1,2]. A one-atom-thick single-layer of carbon was peeled from graphite in 2004 by A. K. Geim and K. S. Novoselov [2]. Graphene is the world’s strongest and thinnest material, and if used with other materials, it enhances the strength of the materials. Thus, graphene is used in automobiles and building materials [3,4]. Graphene is a highly conductive material and is thus used in microelectronic applications and mobile devices [5,6]. Graphene has a high surface-area-to-volume ratio and is thus used for energy storage applications [7,8,9]. Furthermore, graphene is used in coating, sensor, water treatment, and catalyst applications [10,11,12,13,14,15,16]. Because of these extraordinary properties and their applications, researchers were urged to focus on the preparation of graphene. From 3D graphite, 2D graphene was derived by many methods. The methods includes mechanical exfoliation [17], chemical oxidation [18], pyrolysis [19], chemical vapor deposition [20,21], molecular assembly [22], and ultrasonic exfoliation [23]. Among these methods, ultrasonic exfoliation (liquid-phase exfoliation) shows promising results indicating a high yield of graphene [24,25]. At first, by liquid-phase exfoliation, graphite was exfoliated into graphene using high-boiling solvents such as N-methyl pyrrolidone [23,26]. However, this leads to the reaggregation of graphene sheets. These difficulties were solved using surfactants [27]. After this, research focused on the use of appropriate surfactants [23,28,29,30] on graphene surfaces, which can be utilized in specific applications such as coating [31] and the preparation of graphene composites [32]. Recently, we reported stable graphene dispersions in ethanol, isopropyl alcohol, and methanol, using polypyridine- and polystyrene-based block copolymers [33,34,35]. Adhesion studies between graphene surfaces and monomers were investigated using atomic force microscopy [36]. Monomers with nitrogen substituents showed high adhesion values towards graphene surfaces. Thus, nitrogen substituent monomers such as vinyl pyridine were selected to prepare polypyridine-based block copolymers. The prepared polymers were utilized to disperse graphene in different solvents [33,34,35]. In these works, we confirmed that the nitrogen in polymers plays a vital role in interactions with graphene surfaces. Hence, here we utilized heterocyclic *N*-vinyl-2-pyrrolidone (NVP) monomer, which has nitrogen and oxygen atoms. It was assumed that the oxygen atom might also be involved in the interaction with graphene, in addition to the nitrogen atom that might enhance the stability of graphene dispersion. This was believed for stable graphene dispersion with poly-*N*-vinyl-2-pyrrolidone (PNVP). Many reports are available on the fabrication or functionalization of graphene surfaces using NVP [37,38,39,40]. However, many of them include the preparation of graphene dispersions using graphene derivatives, such as graphene oxide and reduced graphene oxide [39,40]. Only a few reports are available on the exfoliation of graphite [37,38], which consists of many steps, including the exfoliation of graphite, the preparation of polyvinyl pyrrolidone, and the preparation of graphene dispersions. Hence, we propose a facile one-step method to prepare graphene/poly-*N*-vinyl pyrrolidone (GPNVP) composite. Graphite powder was directly exfoliated into graphene and stabilized with PNVP by in situ polymerization of the NVP monomer. Further electrochemical studies were carried out for GPNVP composite in the typical three-electrode system. The cyclic voltammetry (CV) measurement of the GPNVP composite was performed at different scan rates (5–500 mV/s) and CV measurements up to 1000 cycles were carried out to reveal the stability of the GPNVP composite.

## 2. Results and Discussion

**Figure 1:**
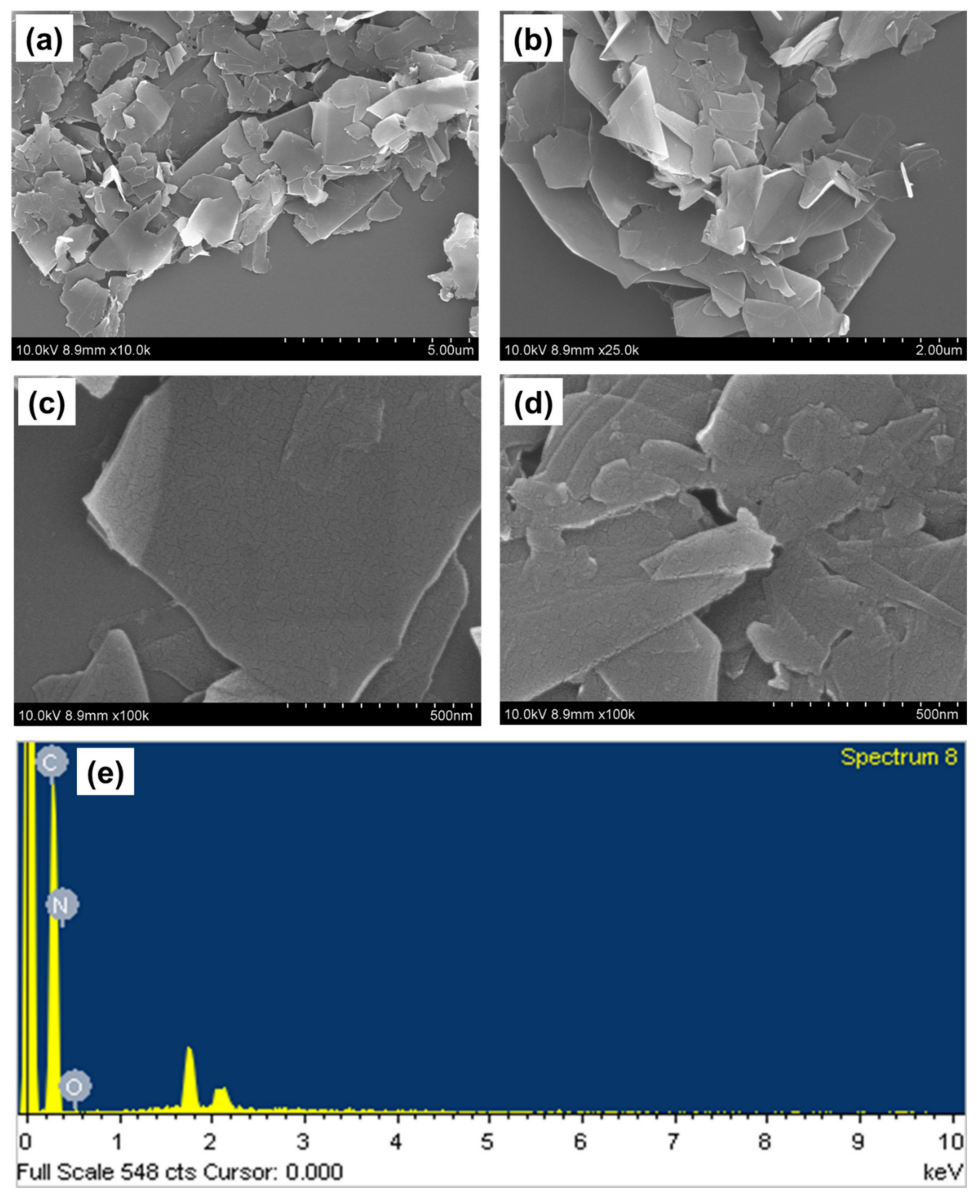
(**a**–**d**) FESEM images of graphene/poly-*N*-vinyl pyrrolidone (GPNVP) composite at different magnifications. (**e**) EDS spectrum of GPNVP composite.

**Figure 2:**
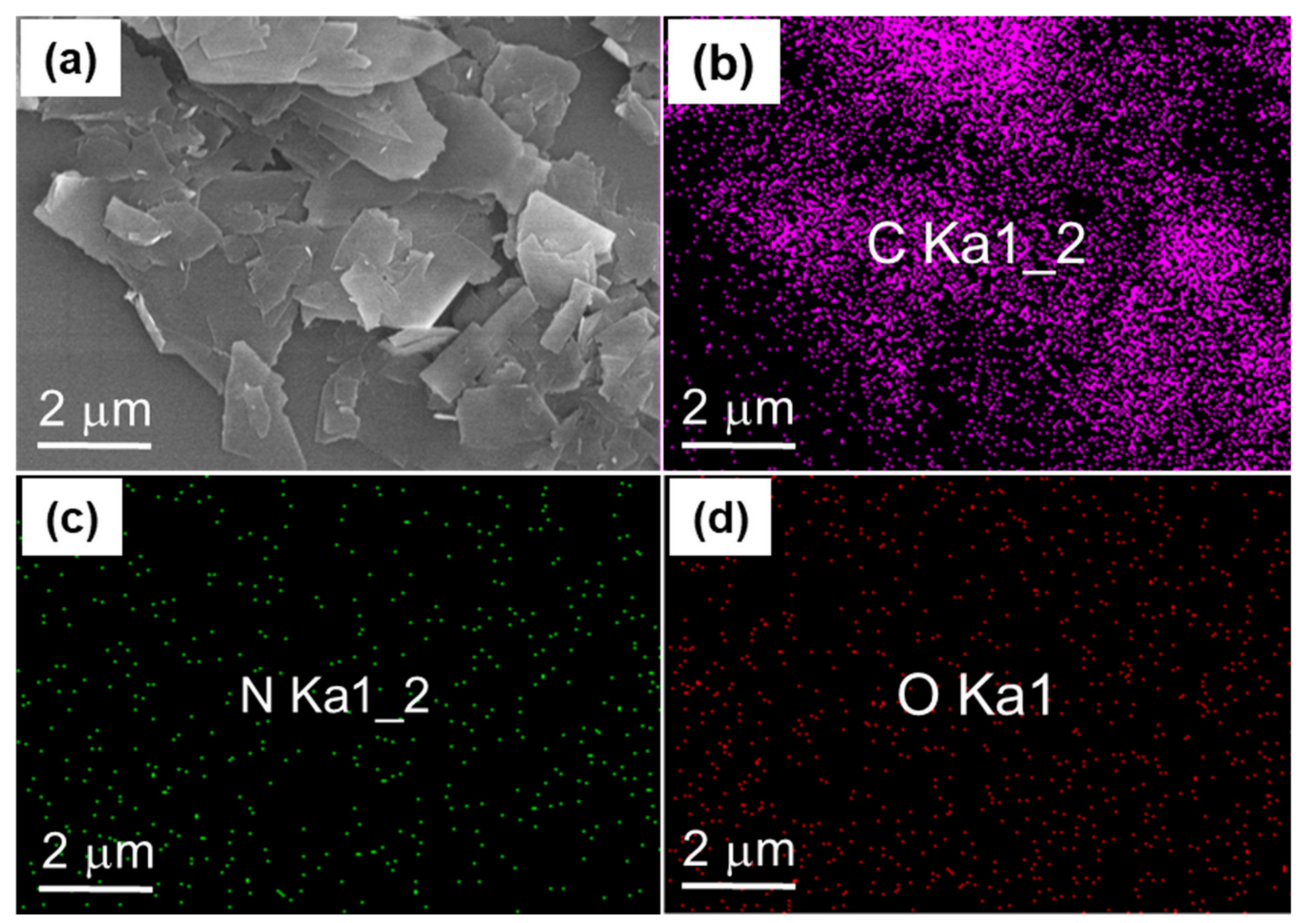
FESEM image of GPNVP composite (**a**) and the corresponding elemental mappings for (**b**) carbon, (**c**) nitrogen, and (**d**) oxygen.

The functionalization of graphene surfaces with PNVP polymer was achieved by in situ polymerization of the NVP monomer. The graphite powder (GP) was directly used for polymerization using the NVP monomer by sonication. GP and the obtained GPNVP composite were characterized using various physicochemical techniques to compare the surface morphologies and chemical compositions and to confirm the successful formation of the GPNVP composite. Field emission scanning electron microscopy (FESEM) and high-resolution transmission electron microscopy (HRTEM) measurements were performed to study the surface morphologies of GP and GPNVP composite. Figures S1 and S2 show the FESEM images of GP and their elemental mapping, respectively. The images reveal the smooth surfaces of GP and a particle size possibly in the few tens of micrometers. The size of the GP was analyzed at a maximum of ~48 μm to a minimum of ~3 μm in lateral size using ImageJ software. The FESEM images at different magnifications reveal the thickness of GP that suggests the presence of many layers of graphene sheets. The energy-dispersive X-ray spectroscopy (EDS) spectrum reveals the presence of carbon (C) and oxygen (O) atoms on the GP surface (Figure S1e). Further, the existence of C and O is confirmed from the elemental mapping images (Figure S2). The surface of GP is covered mainly with C and with a trace amount of O atoms. The FESEM images of the GPNVP composite at different magnifications are shown in Figure 1. Figure 1a,b shows the FESEM images of the GPNVP composite at low magnifications; the images manifest the smooth surface of graphene sheets. The lateral size of graphene sheets will be a few micrometers with a few layers of graphene, which are evident from the images in Figure 1c,d. Using ImageJ software, the sizes of graphene sheets in GPNVP composites were measured at a maximum of ~5 μm to a minimum of ~70 nm in lateral size. Figure 1e depicts the EDS spectrum of the GPNVP composite that divulges the presence of atoms such as C, nitrogen (N), and O. Further, the elemental mapping of the GPNVP composite reveals the presence of C, N, and O atoms (Figure 2).

**Figure 3:**
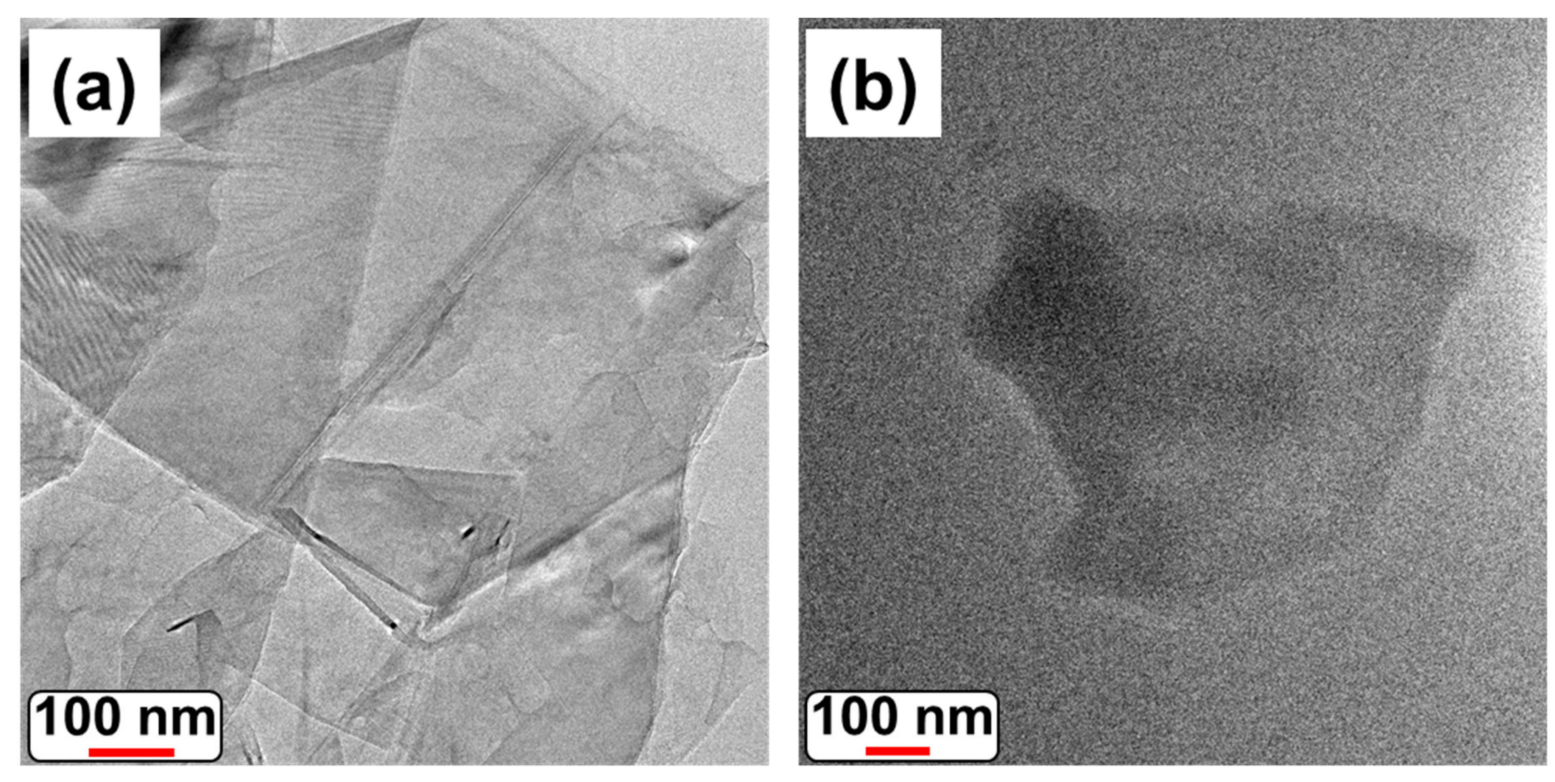
(**a**,**b**) HRTEM images of GPNVP composite.

Further, the surface morphologies of GP and GPNVP composite were studied using HRTEM measurements. Figure S3 shows the HRTEM images of GP that reveal the many layers of twisted graphene sheets (Figure S3a,b). On the other hand, the lattice fringe is clear from Figure 3a, which suggests few-layered graphene in the GPNVP composite. Figure 3b shows a very thin and small-sized graphene sheet. This specifies that in situ polymerization helps in the functionalization of graphene surfaces with PNVP and exfoliates graphite into graphene layers.

**Figure 4:**
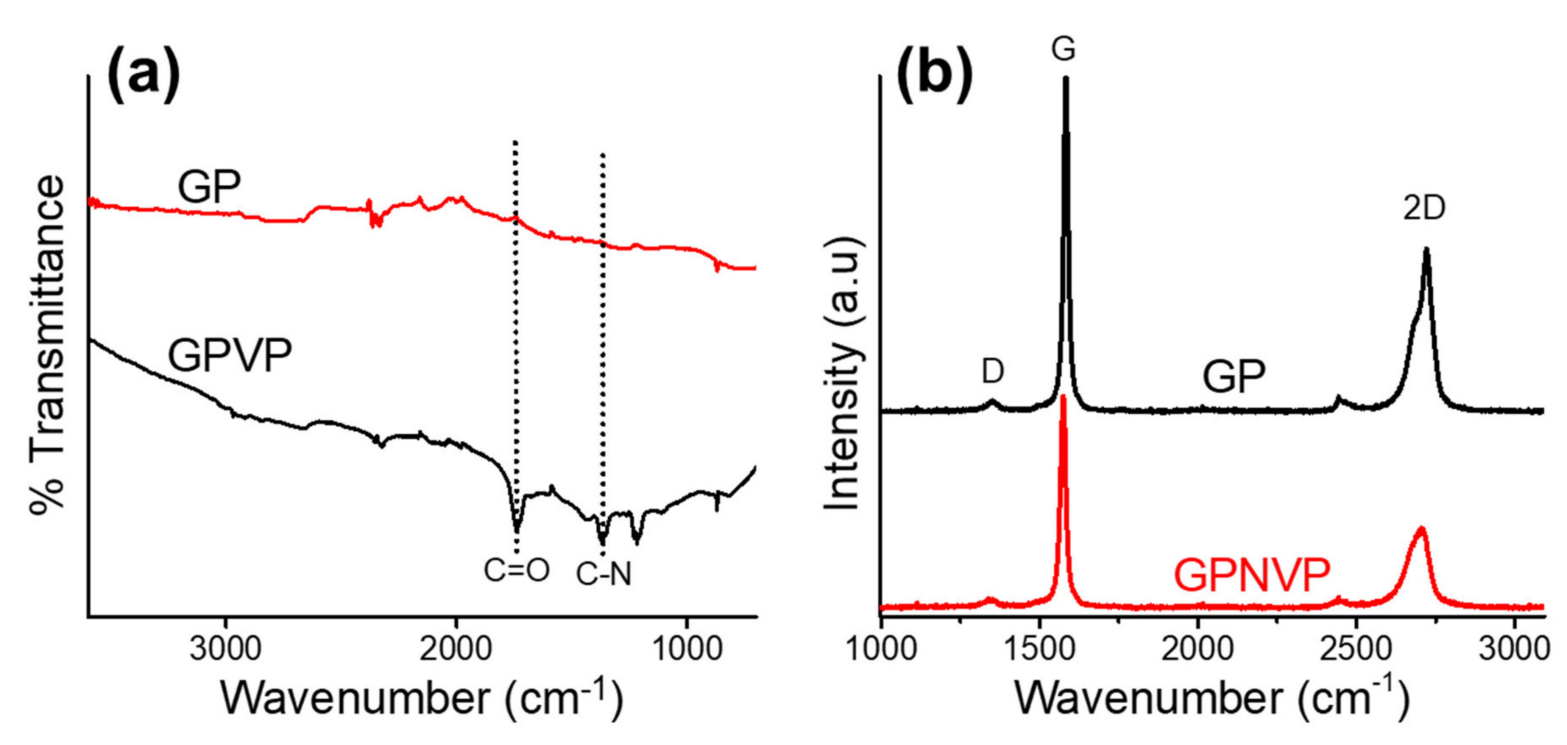
(**a**) Comparison of ATR-FTIR and (**b**) Raman spectra of graphite powder (GP) and GPNVP composite.

Attenuated total reflectance Fourier transform infrared (ATR-FTIR) spectra of GP and GPNVP composite are presented in Figure 4a. The absorption spectra of GP and GPNVP composite divulge the differences between them. The band around 1700 cm^−1^ in GPNVP composite is ascribed to the C=O stretching [41,42], which is not observed in GP. The presence of a weak absorption band around 1600 cm^−1^ in GP and GPNVP composite confirms the presence of sp^2^ C=C stretching of the graphene sheet. This further agrees with the bands around 860 cm^−1^ in both GP and GPNVP composite which are indicative of the out-of-plane bending of the C–H bond from the graphene sheet. The existence of a band around 1400 cm^−1^ reveals the C–N stretching in the GPNVP composite that is absent in GP. The adsorption band around 1200 cm^−1^ might be attributed to the C–C stretching of the polymer backbone. The presence of C=O and C–N stretching from PNVP polymer in the GPNVP composite confirms the successful formation of the GPNVP composite. The Raman spectra for the GP and GPNVP composite in Figure 4b show three characteristic peaks. GP shows the D band around 1347 cm^−1^, G band around 1582 cm^−1^, and 2D band at 2718 cm^−1^. Meanwhile, the GPNVP composite shows the D band around 1342 cm^−1^, G band at 1575 cm^−1^, [43], and 2D band at 2706 cm^−1^. The small D bands in GP and GPNVP composite indicate low or fewer defects. The D band originates from transverse optical phonons around the Brillouin zone corner *K*, breathing modes of the benzene ring in graphene sheets [44]. The first-order Raman scattering by in-plane transverse and longitudinal phonons, which are doubly generated by in-plane vibration modes, are responsible for the G band. Second-order Raman scattering by in-plane transverse optical phonons near the boundary of the Brillouin zone is responsible for the 2D band [45]. Additionally, a small peak around 2440 cm^−1^ for GP and GPNVP composite is assigned as D+D″, which is the combination of D phonon and an acoustic longitudinal phonon D″. D, G, and 2D bands of the GPNVP composite show a slight shift to a lower wavenumber compared to 2D band values of GP. This suggests the exfoliation of graphite into few-layered graphene sheets [46]. The I_D_/I_G_ values correspond to the defect level of composites [47,48]. The I_D_/I_G_ value of GP was calculated as 0.033, while the I_D_/I_G_ value of the GPNVP composite was calculated as 0.036. The slight increase of I_D_/I_G_ values from GP to GPNVP composite suggests that the presence of PNVP on graphene surface is responsible for the slight defects of graphene sheets.

**Figure 5:**
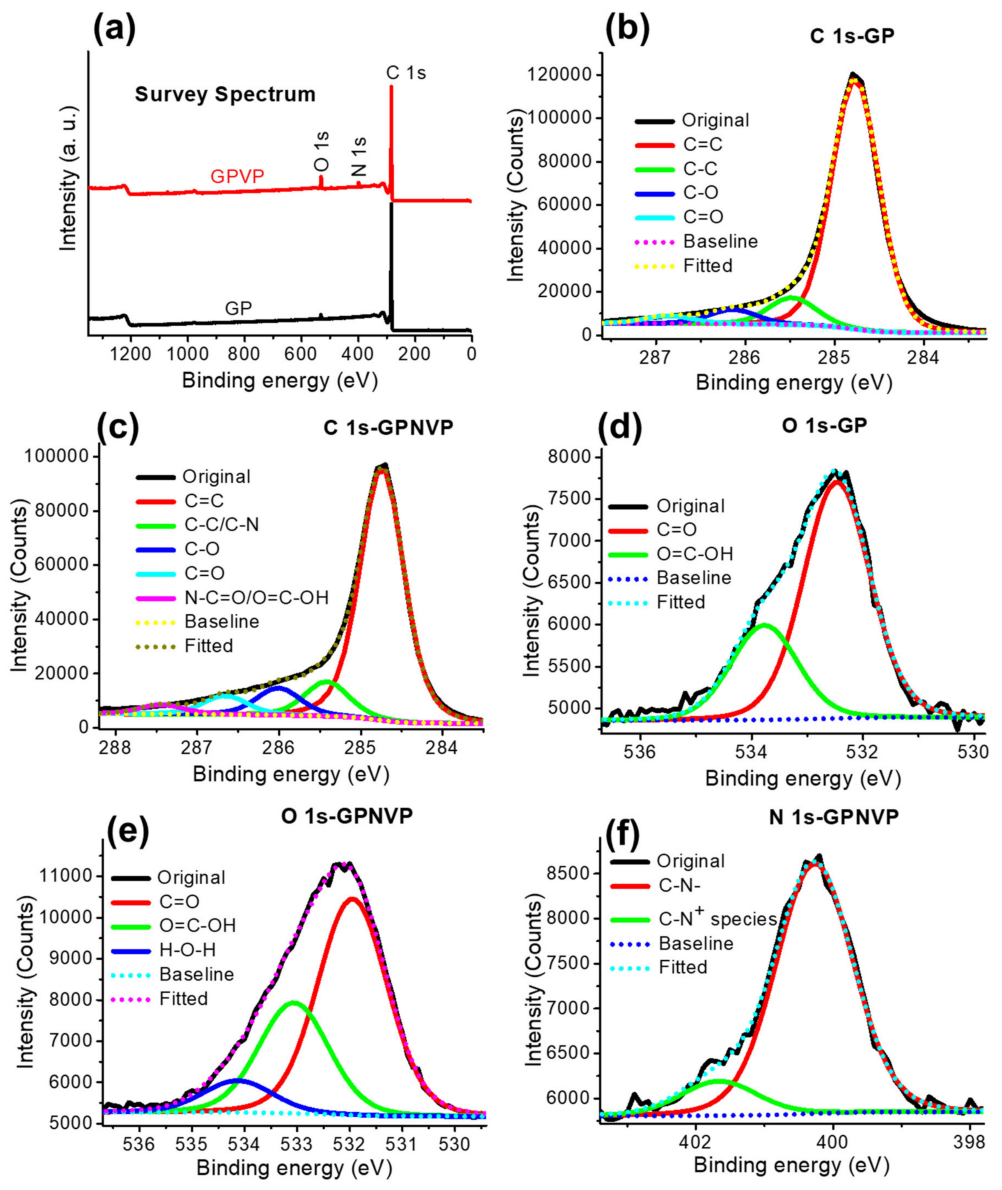
(**a**) XPS survey spectra of GP and GPNVP composite, (**b**) C 1s spectrum of GP, (**c**) C 1s spectrum of GPNVP composite, (**d**) O 1s spectrum of GP, (**e**) O 1s spectrum of GPNVP composite, and (**f**) N 1s spectrum of GPNVP composite.

GP and GPNVP composite were further characterized using X-ray photoelectron spectroscopy (XPS) to investigate the elemental compositions of GP and GPNVP composite. Figure 5a, a survey spectrum, shows the existence of atoms such as O and C in GP and O, C, and N atoms in the GPNVP composite. An additional nitrogen atom in the GPNVP composite is ascribed to the PNVP polymer. Figure 5b,c shows the high-resolution C 1s spectra of GP and GPNVP composite, respectively. The C 1s spectrum of GP shows four peaks at 284.77, 285.45, 286.10, and 286.82 eV that correspond to C=C, C–C, C–O, and C=O, respectively [49]. The C 1s spectrum of the GPNVP composite (Figure 5c) shows five peaks at 284.74, 285.43, 286.06, 286.68, and 287.45 eV that correspond to C=C, C–C/C–N, C–O, C=O, and N–C=O/HO–C=O, respectively [50,51]. A peak around 287.43, which corresponds to N–C=O, originates from PNVP polymer. The high-resolution spectrum (Figure 5d,e) of the O 1s level of GP was deconvoluted into two peaks, and the GPNVP composite was deconvoluted into three peaks. Two peaks of GP at 532.45 and 533.79 eV correspond to C=O and O=C–OH, respectively [50,52,53,54]. Three peaks of the GPNVP composite at 531.96, 533.08, and 534.14 eV are attributed to C=O, O=C–OH, and H–O–H, respectively. The presence of H–O–H (water molecules) in GPNVP composite suggests the hydrophilicity of graphene surfaces in GPNVP composite, unlike the hydrophobic GP. The peak at 531.96 eV can be indexed for the repeated unit of NVP on the graphene surface. The survey spectrum of GP confirms the absence of the N atom. The N 1s peak of the GPNVP composite (Figure 5f) split into two components with binding energies at 400.26 and 401.68 eV. These peaks can be assigned to C–N and positively charged N (C–N^+^) species, respectively [55]. The O 1s level confirms repeated units of NVP on the graphene surface, and the N 1s energy level confirms the presence of C–N and C–N^+^ species in the GPNVP composite. These results confirm the successful formation of the GPNVP composite. Moreover, the relative atomic ratios of C and O in GP and GPNVP composite were calculated as 39.83 and 16.87, respectively. The decrease of C/O ratio in the GPNVP composite when compared to that in GP indicates the successful functionalization of PNVP on the GP surface in the GPNVP composite.

**Figure 6:**
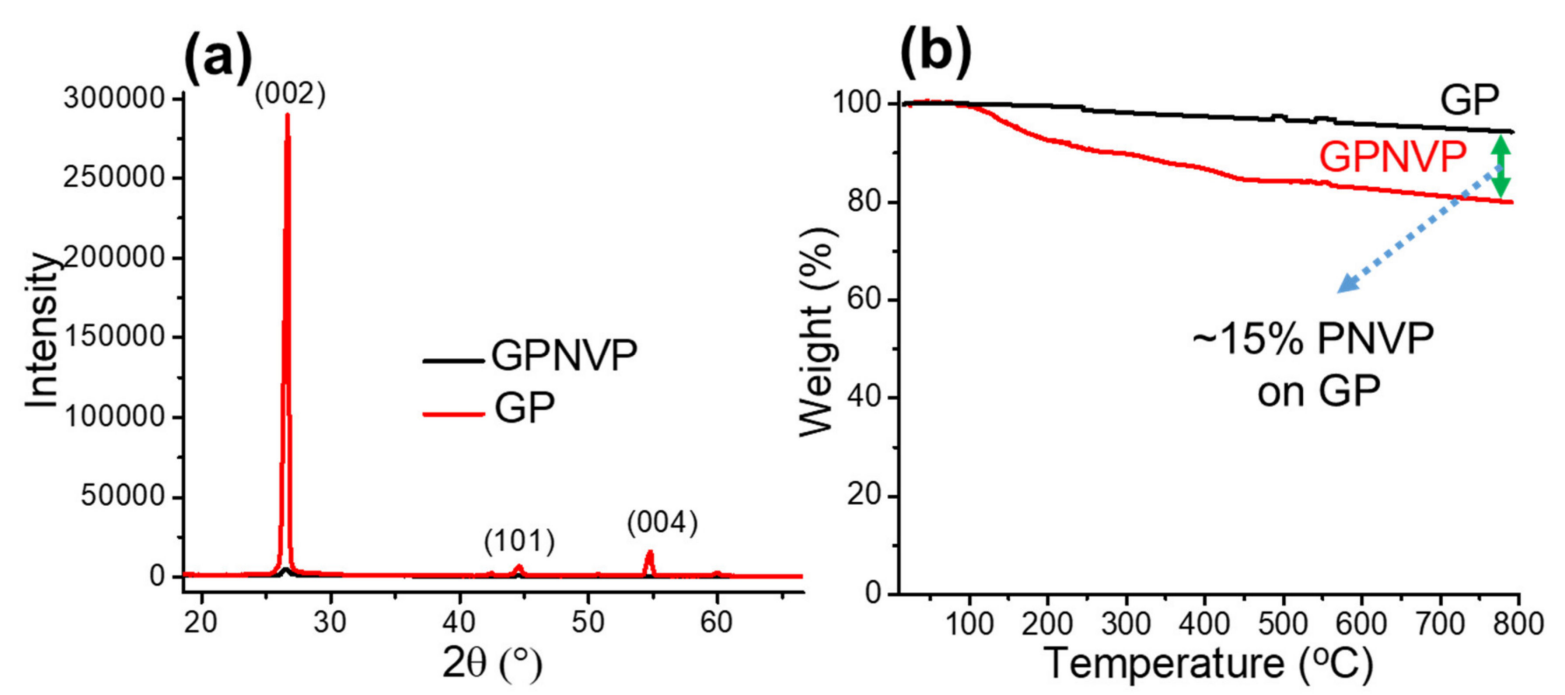
(**a**) XRD patterns and (**b**) TGA curves of GP and GPNVP composite under N_2_ atmosphere.

**Scheme 1:**
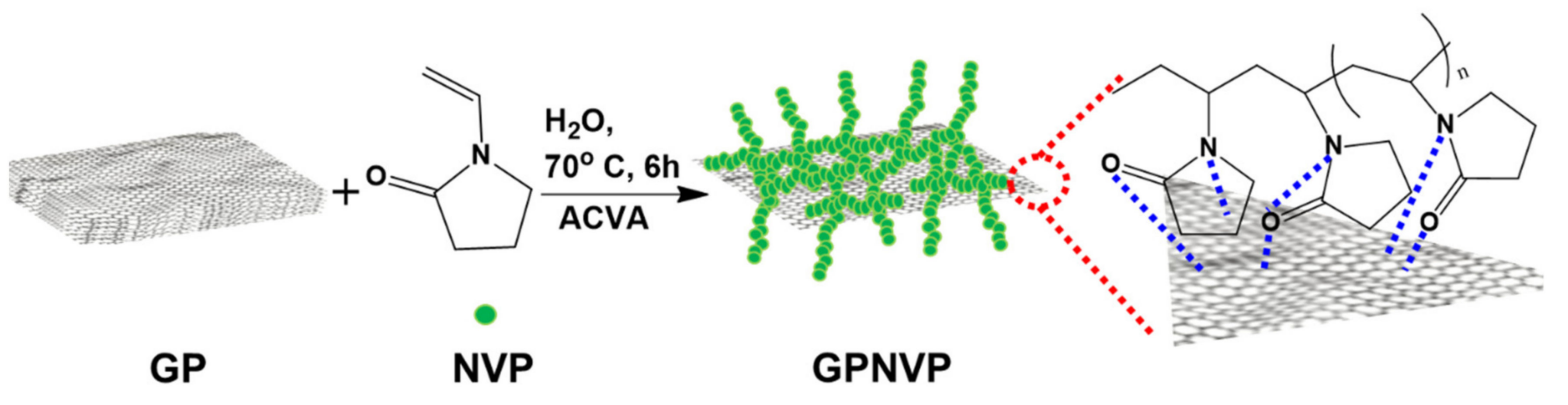
Preparation of graphene surface functionalized by poly-*N*-vinyl-2-pyrrolidone using in situ polymerization.

Figure 6a represents the X-ray diffraction (XRD) patterns of GP and GPNVP composite. The three major characteristic peaks of GP, (002) at 26.65°, (101) at 44.6°, and (004) at 54.79°, are evident from Figure 6a [56,57]. The GPNVP composite displays three peaks: (002) at 26.54°, (101) at 44.58°, and (004) at 54.64°. The GPNVP composite reveals a slight shift in the peaks, broadened diffraction peaks, and a dramatic decrease in the intensities compared to GP. These changes and shifts in peaks suggest the exfoliation of graphene layers in GPNVP composite along with the functionalization of the graphene surface with PNVP [58]. Further, exfoliation is evident from the interlayer distance of the GPNVP composite, calculated as 0.336 nm, which is larger than GP (0.334 nm) [59]. The slight increase in the interlayer distance of the GPNVP composite reveals that the in situ polymerization of NVP monomers on graphene surfaces helps the complete or partial exfoliation of graphite into graphene layers. Figure 6b shows the thermogravimetric analysis (TGA) curves of GP and GPNVP composite. For GP, a slight weight loss above 100 °C, which corresponds to the evaporation of water/moisture, is observed. After this, no significant weight loss is observed, leaving behind about 97% mass. The GPNVP composite reveals a weight loss above 100 °C which contributes to adsorbed moisture or water molecules in the GPNVP composite. The degradation of PNVP starts at a temperature of ~350 °C and proceeds up to 420 °C with weight loss of 84% [60]. In the final step, above 420 to 570 °C, the complete degradation of PVP takes place with weight loss of 83%, leaving around 80% of the carbon residue [61,62]. From this TGA measurement, the amount of PNVP functionalized on graphene surfaces in GPNVP composite is calculated to be approximately 15%. To check the reproducibility of the GPNVP composite, the composite was prepared by adopting the same procedure. Sizes of the graphene sheets in the GPNVP composite were in the error range, and the TGA analysis showed ~15% of PNVP in the GPNVP composite. These measurements confirm the reproducibility of the GPNVP composite. This further supports the functionalization of graphene surfaces with PNVP polymer, the successful formation of the GPNVP composite, and shows good agreement with other results obtained from FESEM, HRTEM, FTIR, Raman, and XPS studies. Further, to explore the dispersion stabilities, photographic images were taken at different time intervals (Figure S4): immediately after the preparation of dispersion, after 1 h, and after 1 month. These figures reveal the stable GPNVP dispersion lasts for at least a month. Significant differences were not observed between the pictures. All analyses confirm the successful formation of the GPNVP composite and the existence of heteroatoms such as C, O, and N. Scheme 1 reveals the interaction between PNVP and graphene surfaces in a GPNVP composite. The stable dispersion is attributed to the interaction of nitrogen atoms from PNVP [34,36]. Additionally, oxygen atoms in PNVP might be involved in the interaction with graphene surfaces and results in stable dispersion for a long time. The existence of heteroatoms and their involvement in the interaction with graphene surfaces is suggested for electrochemical studies.

**Figure 7:**
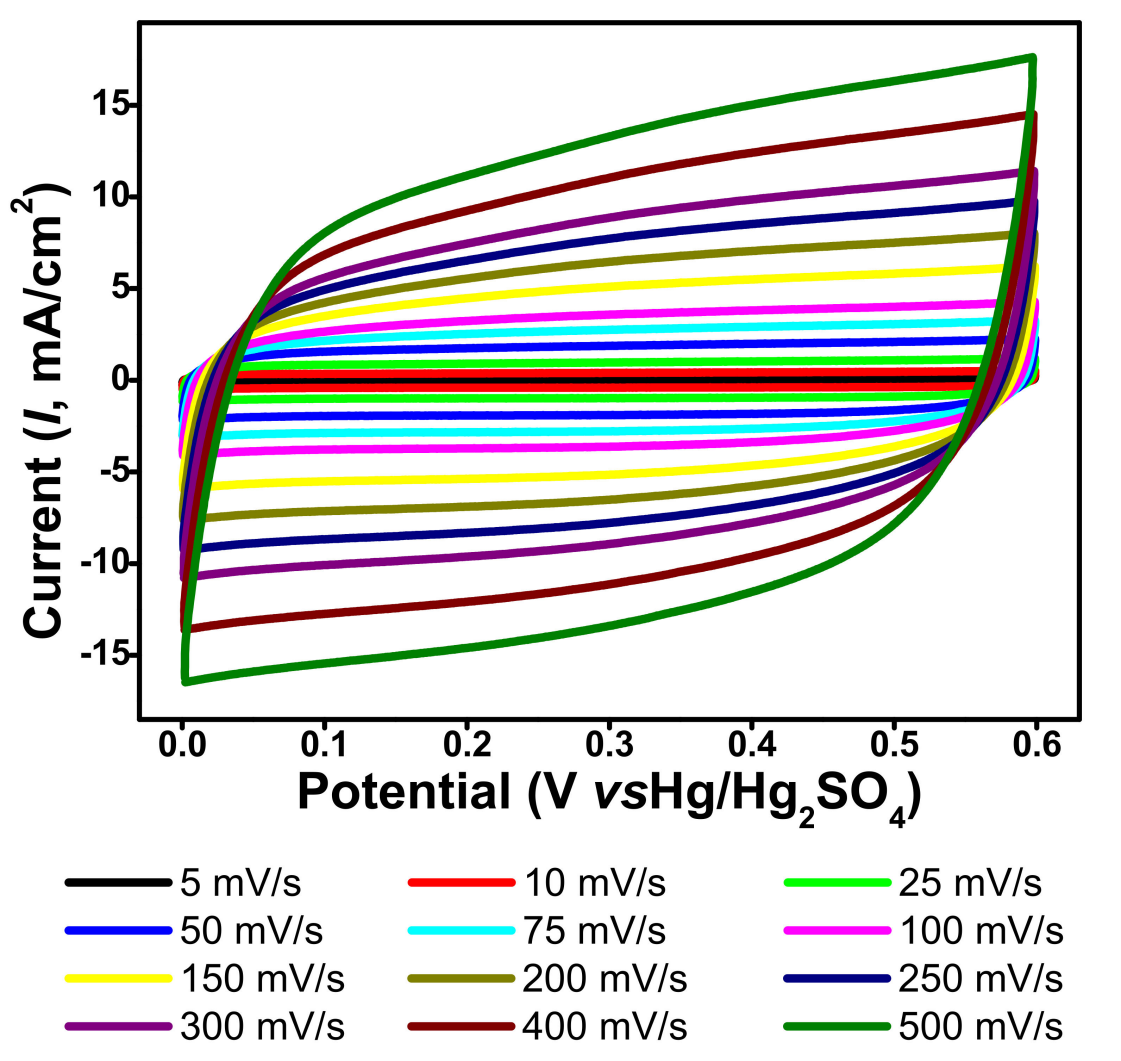
Cyclic voltammograms of GPNVP composite at various scan rates (5–500 mV/s) within the potential range from 0.0 to 0.6 V (vs. Hg/Hg_2_SO_4_) in an aqueous 1 M H_2_SO_4_ electrolyte.

Finally, the cyclic voltammetry (CV) measurement of the GPNVP composite was performed in the typical three-electrode system in an acidic environment to analyze the electrochemical behavior of the resulting GPNVP composite. Figure 7 displays the CV curves of the GPNVP composite at different scan rates of 5, 10, 25, 50, 75, 100, 150, 200, 250, 300, 400, and 500 mV/s within the potential range from 0.0 to 0.6 V (vs. Hg/Hg_2_SO_4_) in an aqueous 1 M H_2_SO_4_ electrolyte. The CV loops of the GPNVP composite show a typical rectangular shape of carbon material without obvious oxygen and hydrogen evolution peaks at all scan rates, indicating the ideal electric double-layer capacitive behavior and good charge propagation within the GPNVP electrode [63,64,65]. The GPNVP electrode displays the obvious increments in the current with an increase in the scan rates from 5 to 500 mV/s. This suggests a good rate capability and good ion transport in the GPNVP electrode [66]. Moreover, it is interesting to note that the GPNVP electrode still maintains a nearly rectangular CV shape even at 500 mV/s. This result suggests that the GPNVP composite has excellent rate capability with the capacitive property. Besides, the CV measurement of the GPNVP composite was performed at a scan rate of 50 mV/s up to 1000 cycles and the corresponding results are shown in Figure S5. The shape and area of the CV curves are comparable even after 1000 cycles (Figure S5), which demonstrates the good rate capability and good electrochemical resistance of the GPNVP composite after many cycles [67]. Moreover, these results suggest that the resulting GPNVP composite electrode could be ideal for various electrochemical applications.

Figure S6 shows the electrochemical impedance spectroscopy (EIS) plot of the GPNVP composite electrode in an aqueous 1 M H_2_SO_4_ electrolyte at room temperature. The EIS Nyquist plot of the GPNVP electrode shows lower solution resistance (2.7 Ω/cm^2^). The smaller distorted semicircle (arc) in the high-frequency region (inset of Figure S6) is due to the double-layer capacitance and charge transfer resistance [68]. The straight line parallel to the *y*-axis with a tilt toward the *x*-axis, observed in the low-frequency regions, is attributed to ion transport through the GPNVP electrode. Moreover, the EIS reveals the high-capacitance behaviors of the GPNVP electrode because of the porous structure of the GPNVP composite. Therefore, the prepared GPNVP composite could be unique for high-powered supercapacitor applications.

## 3. Summary and Conclusions

In this work, we have stabilized graphene surfaces with PNVP and exfoliated graphite to few-layered graphene by in situ polymerization of NVP. The prepared GPNVP composite was characterized well by various analyses, and the results were compared to GP. Surface and elemental mapping by FESEM reveals the uniform distribution of PNVP over the graphene surface, and thin layers of graphene sheets were confirmed from FESEM and HRTEM analyses. ATR-FTIR and XPS revealed the presence of PNVP on the graphene surface in the GPNVP composite. XRD and Raman studies of GPNVP suggest the partial exfoliation of graphite into graphene sheets. Thus, all the physicochemical techniques confirm the successful formation of GPNVP and functionalization of PNVP on the graphene surface. Further TGA confirmed the existence of 15% of PNVP in the GPNVP composite. Besides, the electrochemical performance of the prepared GPNVP composite as an electrode material was investigated by CV measurements within the potential range from 0.0 to 0.6 V (vs. Hg/Hg_2_SO_4_) and EIS study in an aqueous 1 M H_2_SO_4_ electrolyte. The fabricated GPNVP composite electrode exhibited good rate capability and reversibility at a high scan rate of 500 mV/s. Moreover, it is interesting to note that the GPNVP electrode possesses long cycle life and maintains a nearly rectangular CV shape even after 1000 cycles. The aforesaid results suggest that the prepared GPNVP composite is suitable for practical applications, including in various electrochemical fields. The present study has a limitation towards the determination of the molecular weight of polymers that functionalized on the graphene surface, which will be considered in the future. However, this report will focus attention on the preparation of a stable graphene composite in a facile way and directs for the usage of composites in different applications.

## 4. Materials and Methods

### 4.1. Materials

GP and 4,4′-azobis(4-cyanovaleric acid) (ACVA, ≥98.0%) were obtained from Sigma-Aldrich and used as received. NVP ≥ 99% contains sodium hydroxide as an inhibitor. *N*-Methyl-2-pyrrolidone (NMP, 99%), polyvinylidene fluoride (PVDF, MQ100), and sulfuric acid (H_2_SO_4_, 95%) were purchased from Sigma-Aldrich. Distilled and deionized (DI) water was used in all experiments. In situ polymerization of the NVP monomer on GP was carried out using a bath sonicator (40 kHz, 110 W, BRANSON 3800, CT, USA). Bare carbon cloth (CC) was purchased from FuelCellsEtc (College Station, TX, USA) and used for electrochemical studies.

### 4.2. Preparation of Graphene Dispersion by In Situ Polymerization of NVP on GP Surface

The GP surface was stabilized with PNVP by the in situ polymerization of NVP monomer, as shown in Scheme 1. For this, 350 mg (3.14 mmol) of NVP monomer and 44.1 mg (0.15 mmol) of ACVA as an initiator were placed in a 70 mL glass vial. To this, 175 mg GP and 70 mL of degassed DI water were added and purged with nitrogen for 30 min. After 30 min, the glass vial was sealed carefully and sonicated in a bath sonicator. The mixture was sonicated and the bath temperature was maintained at 70 °C, for the polymerization of NVP, for 7 h. Subsequently, the GPNVP dispersion was centrifuged (VS-18000M, VISION Scientific Co. Ltd., Yuseong-Gu, South Korea) for 15 min at 5000 rpm. The unreacted monomers and free polymers were washed out from the prepared GPNVP composite by centrifugation. The supernatant layer was decanted and dried for further use.

### 4.3. Characterization Methods

Pristine GP and GPNVP composite were characterized using various physicochemical techniques. ATR-FTIR spectra were recorded in transmittance mode on a Perkin Elmer Spectrum Two in the wavenumber range from 700 to 3700 cm^−1^. ATR-FTIR was used to evaluate the chemical compositions and to confirm the formation of GPNVP. The Raman spectrum was recorded on an XploRA Micro-Raman spectrophotometer (Horiba, Palaiseau, France) with a range between 1000 and 3200 cm^−1^ at the Core Research Support Center for Natural Products and Medical Materials of Yeungnam University. FESEM with EDS was used to evaluate the surface morphology of the composites. FESEM with EDS measurements were carried out using Hitachi S-4800 equipped with EDX at an accelerating voltage of 10 kV. Further, the morphologies were confirmed from HRTEM images that were obtained from Tecnai G2 F20 S-TWIN with an operating accelerating voltage of 120 kV. The XPS spectra were recorded using a K-Alpha (Thermo Scientific, Seoul, South Korea). CasaXPS software was used for the deconvolution of the high-resolution XPS spectra. XRD studies were carried out using a PANalytical X’Pert^3^ MRD diffractometer with monochromatized Cu Kα radiation (λ = 1.54 Å) at 40 kV and 30 mA and were recorded in the range from 10 to 70° (2*θ*). TGA measurement was carried out on an SDT Q600 with nitrogen atmosphere over 20 (room temperature) to 800 °C with 10 °C/min.

### 4.4. Fabrication of Working Electrode and Cyclic Voltammetry Measurements

The prepared GPNVP composite was used for the fabrication of the working electrode. To fabricate the working electrode, GPNVP composite and PVDF in the ratio 95:5 were ground well in an appropriate amount of NMP to make a homogeneous paste. The resulting homogeneous paste was coated on the conducting CC with an area of 1 cm^2^ by the drop-casting method, after which the electrode was kept at 100 °C in a hot-air oven for 48 h to dry the electrode. After fabrication, the modified working electrode (GPNVP/CC) was examined using CV in a typical three-electrode system. The CV studies were performed on a CorrTest-CS350 electrochemical workstation in an aqueous 1 M H_2_SO_4_ electrolyte. A commercial Hg/Hg_2_SO_4_ (Sat. K_2_SO_4_) electrode was employed as a reference electrode and a platinum plate (1 cm^2^) was used as a counter electrode. The CV measurements were carried out within the potential ranges between 0.0 and 0.6 V at the different scan rates of 5, 10, 25, 50, 75, 100, 150, 200, 250, 300, 400, and 500 mV/s. EIS measurements were performed in the frequency range of 0.01–100 kHz with an alternating current amplitude of 5 mV. The electrochemical test was conducted at room temperature.

## Data Availability

The data presented in this study are available on request from the corresponding author.

## Supplementary Materials

The following are available online, Figure S1: FESEM images of GP surfaces at different magnifications and its EDS spectrum, Figure S2: (a) FESEM image of GP surface and their corresponding elemental mapping (b) carbon and (c) oxygen, Figure S3: HRTEM images of GP with different magnifications, Figure S4: Photographic images of GPNVP composite dispersions at different time intervals, immediately, after 1 h and 1 month, Figure S5: Cycling performance of GPNVP electrode (before and after 1000 CV cycles) at a scan rate of 50 mV/s, and Figure S6: Electrochemical impedance spectrum (EIS) of GPNVP electrode (inset: magnification of EIS) in an aqueous 1 M H_2_SO_4_ electrolyte at room temperature.

## Sample Availability

Samples of GPNVP composite is available from the authors.
