# Supplementary material for: Exfoliation and Noncovalent Functionalization of Graphene Surface with Poly-N-Vinyl-2-Pyrrolidone by In Situ Polymerization
Source: Molecules. 2021 Mar 11;26(6):1534. doi: 10.3390/molecules26061534 (PMC7999643; doi:10.3390/molecules26061534)
Supplement: Supplementary file 1 [file molecules-26-01534-s001.pdf]

Supporting Information

Exfoliation and Non-Covalent Functionalization of Graphene Surface with the Poly-N-Vinyl-2-Pyrrolidone by *In Situ* Polymerization

Suguna Perumal[‡], Raji Atchudan[‡,*], Thomas Nesakumar Jebakumar Immanuel Edison[‡], Jae-Jin Shim[*], Yong Rok Lee[*]

School of Chemical Engineering, Yeungnam University, Gyeongsan 38541, Republic of Korea

[‡]Authors contributed equally to this work.

[*]Corresponding authors.

E-mail addresses: atchudanr@yu.ac.kr (R. Atchudan); yrlee@yu.ac.kr (Y.R. Lee); jjshim@yu.ac.kr (J. J. Shim)

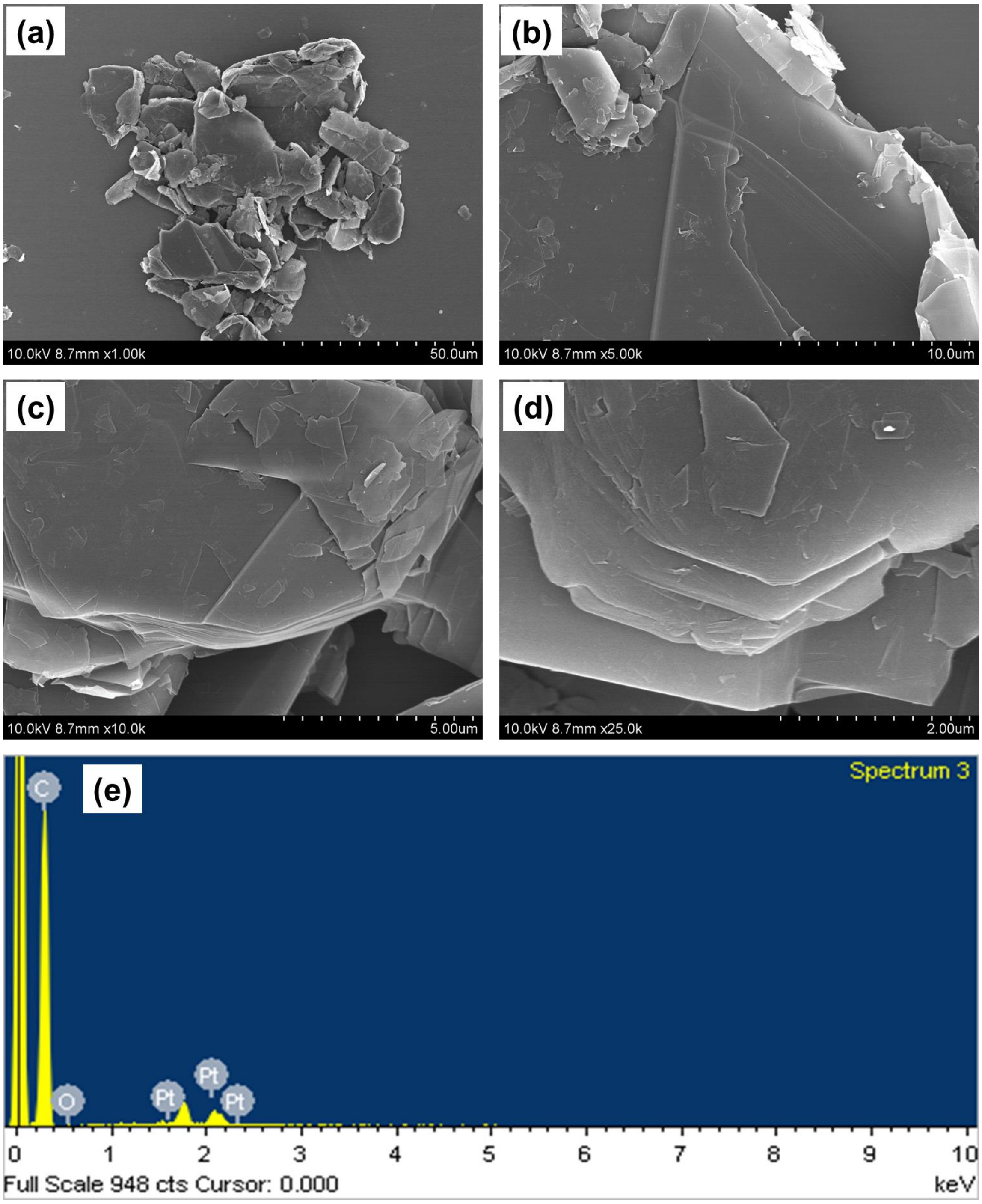

Fig. S1 FESEM images of GP surfaces at different magnifications and its EDS spectrum.

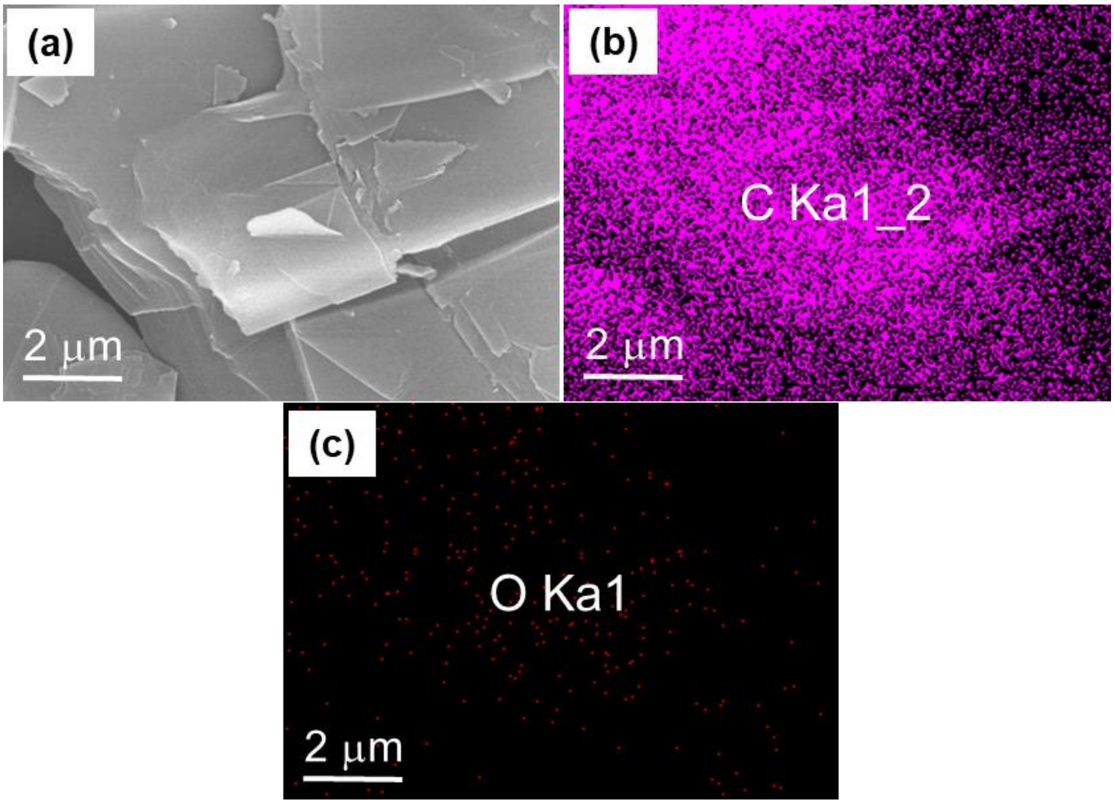

Fig. S2 (a) FESEM image of GP surface and their corresponding elemental mapping (b) carbon and (c) oxygen.

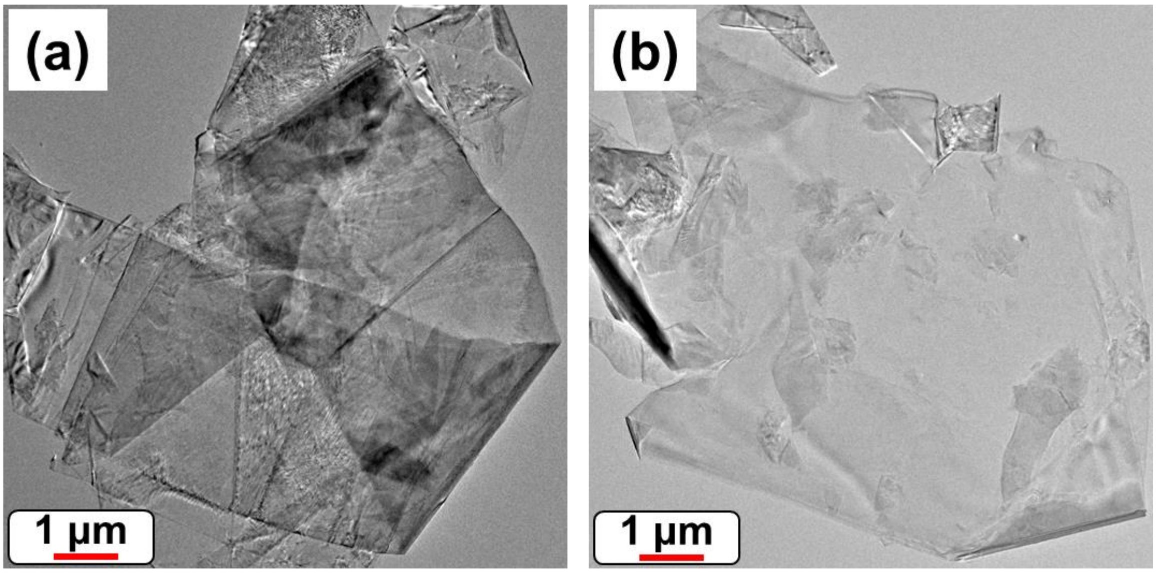

Fig. S3 HRTEM images of GP with different magnifications.

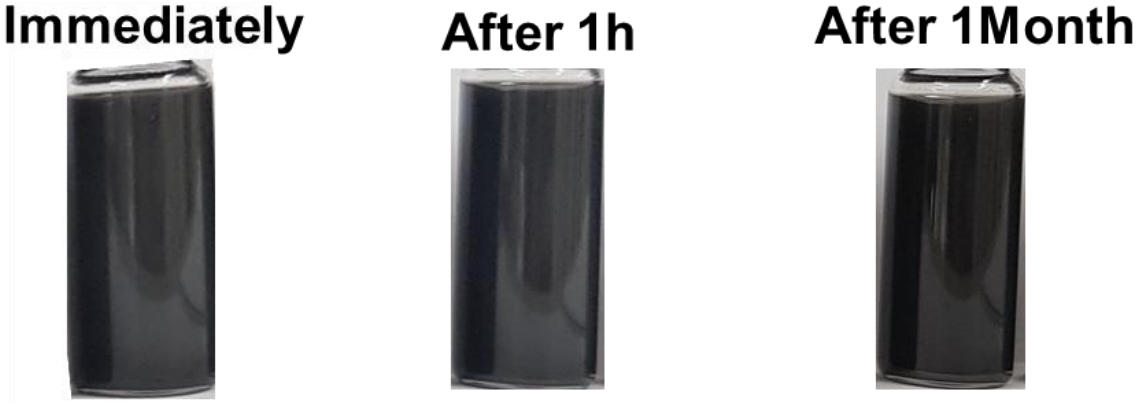

Fig. S4 Photographic images of GPNVP composite dispersions at different time intervals, immediately, after 1 h and 1 month.

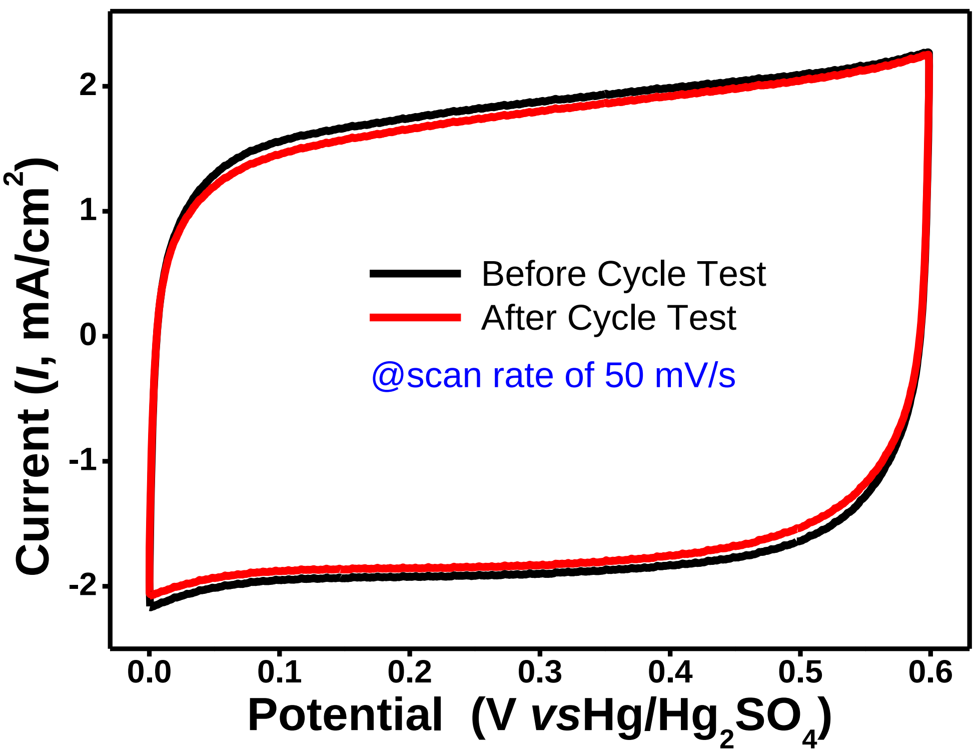

Fig. S5 Cycling performance of GPNVP electrode (before and after 1000 CV cycles) at a scan rate of 50 mV/s.

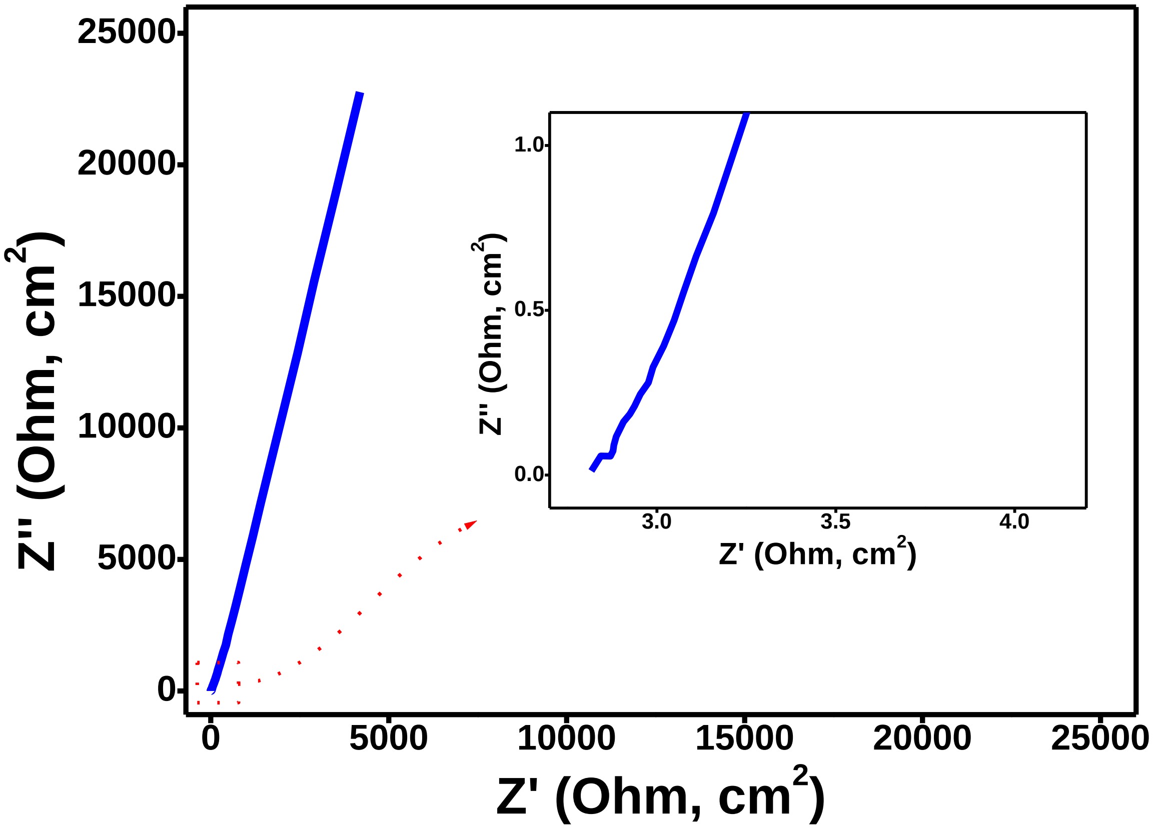

Fig. S6 Electrochemical impedance spectrum (EIS) of GPNVP electrode (inset: magnification of EIS) in an aqueous 1 M H_2SO_4 electrolyte at room temperature.
